# How does variation in total and relative abundance contribute to gradients of species diversity?

**DOI:** 10.1002/ece3.9196

**Published:** 2022-08-17

**Authors:** Thore Engel, Shane A. Blowes, Daniel J. McGlinn, Nicholas J. Gotelli, Brian J. McGill, Jonathan M. Chase

**Affiliations:** ^1^ Institute of Computer Science Martin Luther University Halle‐Wittenberg Halle (Saale) Germany; ^2^ German Centre for Integrative Biodiversity Research (iDiv) Leipzig Germany; ^3^ Department of Biology College of Charleston Charleston South Carolina USA; ^4^ Department of Biology University of Vermont Vermont USA; ^5^ School of Biology and Ecology, and Senator George J. Mitchell Center of Sustainability Solutions University of Maine Orono Maine USA

**Keywords:** Hill numbers, Hurlbert ENS, latitudinal diversity gradient, more‐individuals hypothesis, passive sampling, rarefaction

## Abstract

Patterns of biodiversity provide insights into the processes that shape biological communities around the world. Variation in species diversity along biogeographical or ecological gradients, such as latitude or precipitation, can be attributed to variation in different components of biodiversity: changes in the total abundance (i.e., more‐individual effects) and changes in the regional species abundance distribution (SAD). Rarefaction curves can provide a tool to partition these sources of variation on diversity, but first must be converted to a common unit of measurement. Here, we partition species diversity gradients into components of the SAD and abundance using the effective number of species (ENS) transformation of the individual‐based rarefaction curve. Because the ENS curve is unconstrained by sample size, it can act as a standardized unit of measurement when comparing effect sizes among different components of biodiversity change. We illustrate the utility of the approach using two data sets spanning latitudinal diversity gradients in trees and marine reef fish and find contrasting results. Whereas the diversity gradient of fish was mostly associated with variation in abundance (86%), the tree diversity gradient was mostly associated with variation in the SAD (59%). These results suggest that local fish diversity may be limited by energy through the more‐individuals effect, while species pool effects are the larger determinant of tree diversity. We suggest that the framework of the ENS‐curve has the potential to quantify the underlying factors influencing most aspects of diversity change.

## Graphical abstract

Here, we partition diversity gradients into components of the SAD and abundance using the effective number of species (ENS) transformation of the individual‐based rarefaction curve. Comparing well‐known latitudinal gradients of trees and reef fish, we find that although both groups show similar richness gradients, they differ substantially in the contributions of the more‐individuals effects (86% in reef fish, 41% in trees). Our approach opens new doors for the synthesis of biodiversity trends due to natural and anthropogenic changes.

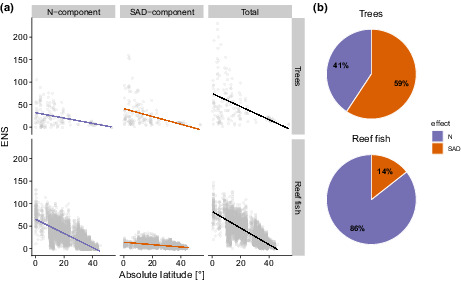

## 1 INTRODUCTION

A fundamental question in ecology is to understand how and why local biodiversity changes from place to place and time to time (Gaston, 2000; Rosenzweig, 1995). Diversity gradients can arise from a number of natural and anthropogenic drivers, and they can inform ecological theory and biodiversity conservation. For example, species richness (i.e., the number of species in a sample) varies along ecological gradients of productivity (Currie, 1991; Mittelbach et al., 2001) and disturbance (Connell, 1978; Miller et al., 2011; Randall Hughes et al., 2007) and along geographic gradients, such as latitude (Fine, 2015; Willig et al., 2003), elevation (Rahbek, 1995), and island size (Kreft et al., 2008). The quantification of diversity gradients from ecological samples is not a trivial problem because diversity is an inherently multidimensional and scale‐dependent quantity that encompasses the occurrences and abundances of multiple species simultaneously and changes with sample size, effort, and spatial scale (Chase et al., 2018). Therefore, species richness usually does not sufficiently capture the nuance underlying any pattern of species diversity.

While the exact drivers and processes shaping diversity gradients are manifold, all of them generally invoke responses in at least one of three broad components of species diversity (Chase & Knight, 2013; He & Legendre, 2002; McGill, 2011): (1) the species abundance distribution (SAD) of a regional species pool (i.e., the total number of species in a region and their relative and absolute frequencies), (2) the total abundance (i.e., the number of individuals [*N*] supported by the environment), and (3) the spatial distribution of species in the region (e.g. intraspecific aggregation and interspecific associations). The interplay of these mutually dependent components determines the shape of the regional species–area relationship and ultimately the diversity of local samples at any spatial scale (Tjørve et al., 2008). Therefore, analyzing diversity in terms of these components can provide deeper insights into the nature of multidimensional biodiversity patterns than analyses of species richness alone (Blowes et al., 2017; Chase et al., 2018), and in turn, this may allow for a better understanding of the processes that shape and maintain diversity gradients at a given scale (Blowes et al., 2020; Gooriah et al., 2021).

For example, a classic hypothesis links species richness gradients to variation in total community abundance, which itself can result from resource and energy gradients, differences in available area or anthropogenic factors (Brown, 2014; Srivastava & Lawton, 1998; Storch et al., 2018; Wright, 1983). The most basic version of this more‐individual hypothesis describes a passive sampling effect, whereby communities with high total abundance simply randomly capture a higher portion the regional species pool than communities with low abundance (Coleman et al., 1982). Such a scenario is qualitatively different from a situation where instead of total community abundance, the SAD of the regional species pool changes along the observed diversity gradient. The evenness and size of the species pool can vary due to various natural and anthropogenic factors that affect species occurrences and abundances in a species‐specific manner, for example, biotic interactions such as competition and predation (Paine, 1974), variation in resource and habitat diversity (MacArthur, 1965; Tilman, 1982), and species specific responses to environmental and anthropogenic filters (Blowes et al., 2020).

To disentangle the components underlying diversity patterns (e.g., SAD and total abundance), it is generally advised to consider several metrics of biodiversity simultaneously because different incidence and abundance‐based diversity metrics (e.g., Hill Numbers, rarefied richness, evenness, and beta‐diversity) capture the aspects of multidimensional diversity change in a complementary manner (Chao et al., 2014; Chase et al., 2018; McGlinn et al., 2019; Roswell et al., 2021). For example, by comparing patterns in observed species richness to those in rarefied richness (i.e., richness standardized for abundances), it is possible to assess whether a diversity gradient is accompanied by more‐individuals effects or changes in the regional species pool (Chase et al., 2018). However, such approaches typically only offer qualitative insights because effect sizes from different diversity metrics are not quantitatively comparable (Dauby & Hardy, 2012). For example, one may find that more‐individual effects seem to play a role for a gradient, but it usually remains unclear exactly what proportion of a diversity gradient can be attributed to variation in total abundance and associated passive sampling effects, and what percentage to changes in the regional SAD (but see McGlinn et al., 2019, 2021).

Here, we present a quantitative dissection of the relative importance of changes in *N* versus changes in the SAD for driving patterns of local species diversity. Effects of aggregation only emerge at larger spatial scales and require spatially explicit data, and we do not address aggregation further here. For our approach, we decompose the total diversity of a sample into two additive components. One component is driven by the SAD and its changes, and the other is driven by the number of individuals (*N*) and associated passive sampling effects. The SAD‐component can be thought of as the sample's expected diversity for a standard number of individuals (*n*), and the *N*‐component is the portion of the observed diversity that is attributable to the fact that a sample exceeds this standard number of individuals (i.e., *N*‐component = total diversity – SAD‐component). Then, we can analyze and compare the changes in the two components (which we call SAD‐effects and *N*‐effects), rather than simply analyzing the total diversity change. To calculate the components, we use the effective numbers of species (ENS) transformation of the rarefaction curve (Dauby & Hardy, 2012), which allows us to express SAD‐ and *N*‐components in the same units of ENS. We illustrate our approach by applying it to two empirical data sets that have strong latitudinal gradients of local species richness (i.e., reef fishes and trees) and show that they emerge from different relative contributions of changes in the regional SAD and in the number of individuals.

## 2 ENS RAREFACTION AND RELATED APPROACHES

Our approach relies on a family of diversity measures that was first introduced as “Hurlbert ENS” by Dauby and Hardy (2012). Here, we use the term “ENS rarefaction” to emphasize that these measures are simply an effective number of species (ENS) transformation of the individual‐based rarefaction (IBR) curve (Hurlbert, 1971). Since ENS rarefaction is one of the lesser‐known, but quite powerful, families of diversity measures, we briefly explain it below and compare it with the related Hill number framework, and the IBR framework that it is based on (see Table 1).

**TABLE 1:** Comparing Hill numbers, individual‐based rarefaction, and ENS rarefaction frameworks for quantifying diversity

	Hill numbers	Individual‐based rarefaction	ENS rarefaction
Symbol	^*q*^ *D*	$S_{n}$	$E_{n}$
Formula	${}^{q}D={\left(\sum_{i=1}^{S}p_{i}^{q}\right)}^{\frac{1}{1-q}}$ Equation (1)	$S_{n}=S-\sum_{X_{i}\geq 1}\frac{\left(\begin{array}{c} N-X_{i}\\ n \end{array}\right)}{\left(\begin{array}{c} N\\ n \end{array}\right)}$ Equation (2)	$S_{n}=E_{n}\left(1-{\left(1-\frac{1}{E_{n}}\right)}^{n}\right)$ Equation (3)
Range	1, *N*	1, *n*	1, ∞
ENS	Yes	No	Yes
Estimation bias	Downward bias for *q* < 2	Unbiased	Unbiased
Description	ENS transformation (“true diversity”) of any diversity index that is a function of $\sum_{i=1}^{S}p_{i}^{q}$ (e.g. Richness (*q* = 0), Shannon (*q* = 1), Simpson (*q* = 2)); Defined as the species richness of a hypothetical perfectly even community that has the same diversity index value as the sample	The expected species richness of a sample of *n* individuals (*n* < *N*)	ENS transformation of $S_{n}$. Defined as the species richness of a hypothetical community that has the same rarefied richness ($S_{n}$) as the sample and infinitely many individuals
Influence of relative abundances	The higher *q*, the lower the influence of rare species	The higher *n*, the higher the influence of rare species	The higher *n*, the higher the influence of rare species
References	Hill (1973), Jost (2006)	Hurlbert (1971), Gotelli and Colwell (2001)	Dauby and Hardy (2012)

*Note*: *S*, observed species richness; $p_{i}$, relative abundance of species i; *q*, exponent that determines the sensitivity to rare species (0 = very sensitive, 2 = not very sensitive); *N*, observed number of individuals in the sample; $X_{i}$ number of individuals of species *i*; ENS, the effective number of species which is the number of equally abundant species that results in the same value of diversity as the sample. To calculate $E_{n}$, Equation 3 can be solved numerically for given values of $S_{n}$ and *n*.

Relating the complementary information given by a set of diversity measures to the diversity components discussed above is challenging because many metrics are sensitive to more than one component (Chase & Knight, 2013). Furthermore, diversity metrics often differ in their numerical ranges and units (i.e., their numerical constraints), and in the degree to which they are affected by passive sampling effects, which in statistics is called estimation bias (Gotelli & Chao, 2013). For example, species richness, which counts all species independent of their abundance, can attain any integer number, and is strongly affected by the number of individuals in the sample. In contrast, Simpson's index, which gives disproportionately high weight to the dominant species of the SAD, ranges between 0 and 1 and is almost unaffected by sample size (i.e., the number of individuals). Although the two metrics hold complementary information on the SAD and passive sampling effects, their different numerical constraints and estimation biases make it difficult to disentangle the two components and compare their effect sizes (Jost, 2006).

The Hill number framework solves the problem of incompatible numerical constraints by converting diversity index values to effective numbers of species (Equation 1 in Table 1). This encompasses all diversity indices that are a function of the term $\sum_{i=1}^{S}{p_{i}}^{q}$(e.g., species richness for q = 0, Shannon index for q = 1 and Simpson's index for q = 2), where the diversity order, *q*, tunes the weight of species abundances $p_{i}$ (Hill, 1973; Jost, 2006; Rényi, 1961). The term ENS refers to the hypothetical number of species that a perfectly even sample would have if it produced the same index value as the real sample. Hence, Hill numbers relieve diversity indices of their numerical constraints by re‐expressing them in units equivalent to that of species richness (Jost, 2006). However, like most diversity metrics, Hill numbers retain a downward estimation bias, whose strength diminishes with increasing values of the diversity order q (Chao et al., 2014). Therefore, differences in Hill number profiles cannot unambiguously be attributed to changes in the regional SAD or changes in total abundance. For example, if ^2^ *D* (corresponding to Simpson's index) is constant along a gradient of interest while ^0^ *D* (i.e., species richness) increases, this pattern can be underlain by a change in the regional SAD (i.e., an increase in the number of rare species), a passive sampling effect (i.e., an increase in total abundance) or both.

**FIGURE 1:**
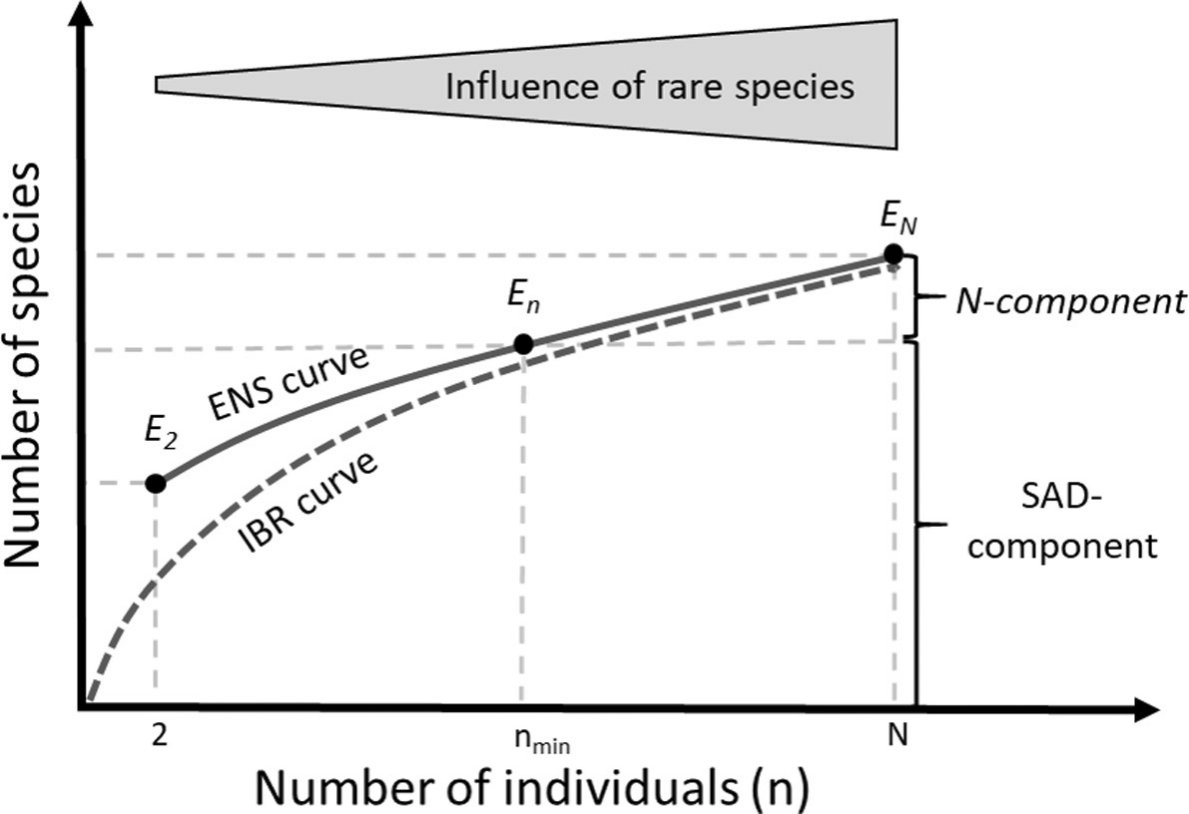
Schematic drawing of an individual‐based rarefaction (IBR) curve and the corresponding effective number of species (ENS) curve. The IBR curve is constrained by the values of *n* (i.e. it is bound to start at the *x* = *y* = 1), whereas the ENS curve is unconstrained on the vertical axis. The ENS value for a standardized number of individuals *E*_*n*_ reflects the “SAD‐component” in our framework. The difference between the total diversity (ENS_*N*_) and the SAD‐component (ENS_*n*_) results from the fact that samples usually exceed the number of individuals *n*_min_ used for standardization. As this portion of the total diversity change reflects abundance variation, we call it “*N*‐component”.

IBR is a framework that explicitly addresses passive sampling effects by expressing diversity in terms of the expected number of species for a standardized number of individuals (Equation 2 in Table 1) (Gotelli & Colwell, 2001; Hurlbert, 1971). The resulting non‐linear scaling relationship between the number of individuals (*n*) and expected species richness (i.e., rarefied richness, *S*_*n*_) is the IBR curve (Figure 1). Rarefied richness estimates are unbiased for random samples, which means that they only respond to changes in the SAD but not to the original number of individuals present in the sample *N*. By varying the reference sample size n, IBR can give more or less influence to species abundances (Gotelli & Colwell, 2001). However, the value of *n* also constrains the numerical range of rarefied richness values. Thus, effect sizes at the base of the IBR curve (representing mostly common species) are not directly comparable to those at higher values of *n* (representing both common and rare species; Dauby & Hardy, 2012). In other words, if we find a species richness gradient to be steeper than a corresponding gradient in rarefied richness, part of the numerical difference has nothing to do with more‐individual effects, but is merely the null expectation from the different numerical constraints of the two metrics.

ENS rarefaction is method that converts the IBR curve into effective numbers of species with consistent numerical constraints along the curve (Figure 1). There is no simple closed‐form equation for ENS rarefaction but Dauby and Hardy (2012) showed that numerical approximation of Equation 3 in Table 1 can be used to convert any *S*_*n*_ value to its corresponding effective number (*E*_*n*_). Again, ENS refers to the number of species in a hypothetical, perfectly even community that has the same rarefied richness as the real community (Dauby & Hardy, 2012). The base of the resulting “ENS curve” (i.e., *E*_2_) is also the ENS transformation of Hurlbert's (1971) unbiased probability of interspecific encounter (*S*_PIE_, Olszewski, 2004), and is equal to an asymptotic estimate of the Hill number ^2^ *D* (Chao et al., 2014; Dauby & Hardy, 2012). It can be interpreted as the number of dominant species in the species pool because being at the base of the curve it gives disproportionately high weight to species with high relative abundances. As *n* increases along the ENS curve, rarer and rarer species influence the diversity estimate until it practically converges onto the observed total species richness, where all species are counted regardless of their abundance (i.e., *E*_*N*_). Increases along the ENS curve are entirely due to the incremental influence of rare species and do not result from variable numerical constraints along the curve. Therefore, the ENS transformation makes it easy to assess relative evenness; random samples from perfectly even communities (i.e., communities without rare species) produce ENS curves that are flat horizontal lines (Dauby & Hardy, 2012). In some sense, ENS rarefaction combines the advantages of Hill numbers and IBR in a single family of diversity measures. It has unconstrained values for all values of *n* and, being a simple transformation of rarefied richness, its values for a reference sample size *n* are only affected by the SAD and not by the actual number of individuals captured in the sample. Therefore, differences in *E*_*n*_ values for a constant *n* can be unambiguously attributed to changes in the SAD, while comparisons between different levels of *n* reflect a quantification of the more‐individuals effect. These properties make ENS rarefaction a useful tool for the decomposition approach we present here.

## 3 ANALYTICAL FRAMEWORK

**FIGURE 2:**
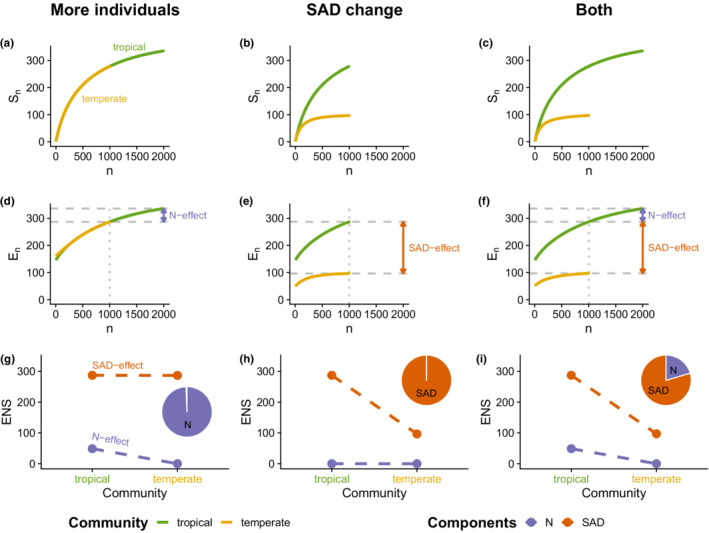
Schematic overview of the analytical framework. Using individual‐based rarefaction curves (a–c) and their conversion to effective numbers of species (ENS) (d–f), diversity change can be dissected into contributions of SAD effects and *N* effects. The columns represent 3 hypothetical scenarios of diversity patterns between a diverse “tropical” and a less diverse “temperate” local community. In first scenario (a, d, g), the difference in diversity results from a passive sampling effect, as the tropical community supports more individuals than the temperate one. In the second scenario (b, e, h), abundance remains constant, but the pattern is underlain by differences in the regional species abundance distribution (SAD, i.e. larger species pool in the tropics). In the third scenario (c, f, i), both abundance and the regional SAD vary between the two communities. Using the ENS conversion, the total diversity of each sample is dissected into a SAD‐component and an *N*‐component (dots in g–i). By examining the difference of the components between the communities, we can quantify the corresponding SAD effects and *N* effects (pie charts in g–i).

Figure 2 illustrates how we use ENS rarefaction to disentangle the diversity components in practice. For this purpose, imagine a latitudinal diversity gradient between a temperate (low diversity) community and a tropical (high diversity) community. We consider three scenarios of how this diversity gradient can manifest in terms of SAD and *N* variation. First, a more‐individuals effect (panels a, d, and g); second, a change in the regional SAD (panels b, e, h); and third, a combination of more‐individuals effect and SAD change (panels c, f and i). The first row of Figure 2 (panels a, b, c) shows the IBR curves corresponding to the 3 scenarios. Panel a depicts the more‐individuals effect, where the tropical community (green) has twice as many individuals as the temperate one (yellow) and therefore samples a larger fraction of its species pool. However, when standardized to a common number of individuals, both communities are expected to yield the same diversity (i.e., the IBR curves follow the same trajectory), which reflects that they are samples from similar regional SADs. Compare that with panel b, where the number of individuals is the same for both communities, but their SADs differ (i.e., the IBR curves have different shapes). In this scenario, the tropical community samples from a larger species pool with a higher number of relatively common species and many more relatively rare species, which results in an IBR curve that is steeper than the temperate one. Finally, panel c represents a scenario where the diversity gradient is underlain by a combination of more‐individuals effects and SAD changes. Not only does the tropical community sample a more diverse SAD but also it harbors a larger number of individuals.

While the IBR curves allow us to qualitatively and visually distinguish the scenarios, they do not directly enable a quantitative decomposition of the observed diversity change into contributions of more‐individuals effects and SAD. Therefore, we apply the ENS transformation to free IBR curves of their numerical constraints. The resulting ENS curves (second row) are similar to the IBR curves in that changes in their shape reflect changes in the SAD, but the start of the curve is no longer constrained. In the more‐individuals scenario (panel d), both communities have the same diversity for any common number of individuals (up to *n* = 1000). Beyond that, the tropical community passively samples additional rare species due its larger sample size (labeled as “*N*‐effect”). In the SAD change scenario (panel e), the ENS transformation reveals that the tropical community has a higher number of relatively dominant species to start with (i.e. *E*_2_), and then accumulates relatively rare species at a higher rate than the temperate community, adding up to the total SADeffect (labeled “SAD‐effect”). The same SAD‐effect can be observed in the combined scenario (panel f), but now the tropical community also has additional rare species due to its higher number of individuals (labeled “*N*‐effect”). As along the ENS curve all values are expressed in terms of effective numbers of species, we can directly compare the magnitudes of the two effects. In this example (panel f), most of the observed diversity change is attributed to changes in the regional SAD (ca. 80%), while the contributions of the more‐individual effect are relatively small (ca. 20%).

To apply this approach to any number of communities, we can partition the total diversity of each community (i.e. *E*_*N*_) into two components: The SAD‐component is simply the ENS for a standard number of individuals (i.e. *E*_*n*_), where *n* is typically the sample size of the smallest community in the gradient. Then, the *N*‐component is the difference between the total diversity and the SAD‐component (i.e. *E*_*N*_ – *E*_*n*_). It reflects the more‐individuals effect with respect to *n* individuals (i.e. how much more diversity does a community have because its sample size exceeds *n*). Now, instead of considering the total diversity (*E*_*N*_), we can analyze these components along the gradient of interest. This is shown in the last row of Figure 2, where the orange and purple dots represent the SAD‐ and *N*‐components of the two example communities. Note that adding up the two components yields the total diversity of the communities. In the first scenario, the diversity change occurs exclusively in the *N*‐component (i.e., a *N*‐effect), while in the second scenario, the diversity change is driven by the SAD‐component (i.e., a SAD‐effect). Finally, in the third scenario both components change at the same time, so that *N* effect and SAD effect add up to the total diversity gradient. By comparing the slopes of the two components along the gradient (dashed lines), we can assess the relative contributions of *N* effects and SAD effects to the observed diversity gradient. The pie charts in Figure 2 illustrate the contributions of SAD effects and *N* effects for each scenario. In the combined scenario (panel i), the SAD effect contributes 80% toward the total diversity gradient while 20% of the diversity change occurs because the tropical community has more than 1000 individuals. In practice, these effect sizes correspond to the regression coefficients of linear models. However, the components could also be modeled as non‐linear functions of continuous predictors. In that case, the contributions of *N* and SAD effects may be variable along the gradient and cannot be summarized as a simple pie chart.

## 4 SIMULATION

To quantitatively examine the behavior of the two components with respect to variation in the SAD and total abundance, we carried out a simulation study using the R package mobsim (May et al., 2018). We simulated spatially explicit Poisson communities (i.e., species had random spatial distributions) with different SADs and total abundances. We assumed lognormal SADs for the simulated communities and parameterized them with different species pools (100, 200, 300, 400, 500 and 600 species) and total abundances (1000, 2000, 3000, 4000, 5000, and 6000 individuals) in a full‐factorial design, using 20 replicates for each factor combination. We then sampled each of the communities with a constant quadrat size corresponding to 20 percent of the total area. Following the approach outlined above, we calculated the SAD‐ and *N*‐components for the samples and examined how they responded to the simulation parameters (i.e., species pool and total abundance). Our simulations show that the SAD‐component responded to changes in the species pool but remained unaffected by total abundance (Figure S1). Conversely, the *N‐*component consistently responded to changes in total abundance and was unaffected by changes in underlying SAD (Figure S2). The findings from these simulations are consistent our theoretical expectations from the IBR curve, and the conceptual example is shown in Figure 2.

## 5 CASE STUDIES: CONTRASTING LATITUDINAL GRADIENTS IN TREES AND REEF FISH

We used our approach to analyze two empirical datasets documenting latitudinal diversity gradients (LDG) in reef fish and trees. The trend of increasing diversity from poles to equator is one of most prominent global biodiversity patterns that occurs in many taxa and at different spatial scales (Fine, 2015; Hillebrand, 2004; Willig et al., 2003). All components, particularly *N* and the SAD, likely vary along the gradient, though how they combine to form the LDG at a given scale, and whether this varies among taxa, is less well known.

For example, *N* is expected to vary with energy‐ or resource availability and, accordingly, the more‐individual hypothesis (MIH) is one of the classic explanations for the LDG (Brown, 2014; Srivastava & Lawton, 1998; Wright, 1983). Historically, the MIH has referred to a collection of different mechanisms by which higher total abundance translates to higher species diversity, including effects on extinction and speciation rates (Evans et al., 2005; Scheiner & Willig, 2005; Storch et al., 2018). However, here we use the term more narrowly to only mean passive sampling effects (Coleman et al., 1982), which is the process by which larger communities (e.g. in the tropics) randomly sample a larger portion of a species pool than small ones (e.g. in temperate regions) (Wright, 1983). Abundance‐related processes that influence extinction (e.g. demographic stochasticity) and diversification rates over the longer term likely alter the SAD and regional species pool, and therefore would be captured by SAD effects in our framework. Indeed, there are a large number of ecological and evolutionary mechanisms that shape and maintain latitudinal gradients in regional SADs. These include differences in time for speciation, environmental stability, species interactions, and niche‐processes (Fine, 2015). While the LDG is generally strongest at larger spatial grains (Hillebrand, 2004), it is largely unknown how such species pool gradients combine with gradients of total abundance to determine local‐scale diversity gradients.

Here, we applied the analytical framework to analyze latitudinal gradients of two publicly available datasets with standardized community surveys: (1) forest trees from the Gentry plot dataset (Gentry, 1988, Phillips & Miller, 2002) and (2) reef fish from the Reef Life Survey (Edgar et al., 2020; Edgar & Stuart‐Smith, 2014). Importantly, both data sets use a fixed sampling effort in terms of plot/transect size for their respective sites. Therefore, latitudinal variation in sample diversity reflects changes in the regional species pool (SAD) as well as natural variation in the observed number of individuals (i.e. more‐individuals effect).

Because our main focus was to illustrate the analytical framework, rather than an exhaustive analysis of these data sets, we reduced both data sets into one latitudinal “slice” to minimize other well‐known confounds, such as biogeographic factors, that influence the magnitude of the gradient. For trees, we focused on the plots located in the Americas, so as to avoid the potential influence of continent on tree diversity (Qian & Ricklefs, 2000). And for the reef fish, we only included surveys from the Indo‐Pacific area where diversity is highest, and biogeographic effects (e.g., distance from diversity center) were minimized (Blowes et al., 2017). For both data sets, we excluded sites with fewer than 20 individuals (we also used different cutoff‐levels to test the robustness of our results). Figure S3 shows the geographical location of samples included in our analyses.

After selecting the sites, we dissected the observed diversity of each sample into the SAD‐component and the *N*‐component, assuming a reference sample size of *n* = 20. To do this, we calculated the observed richness and the rarefied richness (*S*_*n*_) for *n* = 20 and derived the corresponding ENS values using Equation 3 in Table 1 (i.e. *E*_*N*_ and *E*_*n*_, respectively). *E*_*n*_ represents the SAD‐component. The difference between *E*_*N*_ (total diversity) and *E*_*n*_ (SAD‐component) is the diversity component that results from the changes in *N* or the more‐individuals effect (*N*‐component). We then modeled the two components along the latitudinal gradient using simple linear models with absolute latitude as the independent variable, and the SAD and *N*‐components as dependent variables. We used the regression coefficients (or slopes) as the effect sizes for the respective components. Since our partitioning framework is additive and models are linear, the effect sizes (i.e. slopes) of the two components add up to the effect size (i.e. slope) of the total diversity gradient.

**FIGURE 3:**
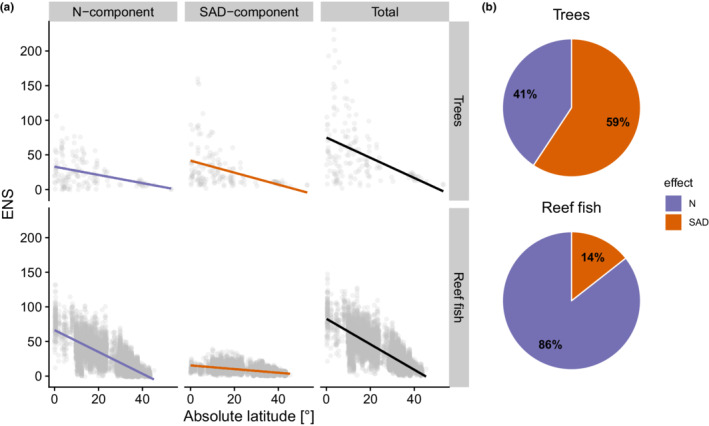
Latitudinal diversity gradients of trees and reef fish. (a) *N*‐component, SAD‐component, and total diversity. Lines represent linear model fits. (b) Relative contributions of *N*‐effects and SAD‐effects toward total diversity gradient, quantified as the corresponding slopes in (a).

Both trees and reef fish showed similar slopes along their respective latitudinal gradient for the overall richness gradient, but they differed in how the underlying component contributions changed along the gradient (Figure 3). The trees had a relatively large SAD effect; that is, even when the number of individuals was standardized, the diversity gradient remained quite strong. This suggests that the diversity gradient is mostly underlain by changes in the species pool and associated patterns of commonness and rarity (i.e., the SAD). Nonetheless, the *N*‐effect also contributed to the total diversity gradient, as total tree abundance tended to increase as absolute latitude decreased. In contrast to the trees, the reef fish diversity gradient was strongly dominated by the *N*‐effect. For a standardized number of individuals, the fish diversity gradient was relatively weak (see SAD‐component). This reflects that species rich reef fish communities are often dominated by a few species, the number of which does not vary strongly along the gradient. For a constant sample size, the many rare species in diverse fish communities have little weight in the diversity estimate. That is, they mostly affect the diversity for communities with more individuals and are captured more‐individual effect.

The contrasting results between fishes and trees could reflect biological differences of the two groups. Fish move in a three‐dimensional space, which allows for much stronger gradients in total abundance. In forests, on the other hand, stem density is likely more strongly limited by available space. This suggests that for forests, community assembly processes change more strongly along the gradient, leading to communities with high relative evenness in the tropics (Ulrich et al., 2016). This is reflected in the strong SAD‐effect. Conversely, the schooling nature of some tropical fishes allows for the dominance of a few species. Additionally, the number of dominant fish species does not vary strongly along the gradient, whereas the number of rare species (which are affected by sampling effects) does. Hence, we find the large *N*‐effect in fishes.

## 6 DISCUSSION

In this paper, we have outlined a quantitative approach for decomposing local diversity change into contributions of changing SADs and more‐individual effects. Using two latitudinal gradients that have similar patterns of species richness, but very different kinds of diversity change, we illustrated the utility of this approach. For trees, a major part of the gradient was attributable to changes in the dominant part of the SAD (59%). Whereas, for reef fishes the diversity gradient was mostly underlain by more‐individual effects (86%). Our case study shows that our approach has great potential for quantitative synthesis studies that analyze the heterogeneity in seemingly general diversity patterns (such as the LDG).

It is not a new idea to describe the diversity components using different metrics derived from the IBR curve (e.g. S_PIE_, S_n_, S, N) (Chase et al., 2018; Hurlbert, 1971; McGlinn et al., 2019; Olszewski, 2004). However, it has been difficult to quantitatively combine the lines of evidence described by multiple metrics, as the corresponding effect sizes are usually not directly comparable. The novelty of our approach is that it uses the common currency of effective numbers of species to decompose the diversity of a sample into a SAD‐component and a *N*‐component that are directly comparable. Whilst deriving our approach, we also shed light onto the commonly overlooked diversity framework of ENS rarefaction (Dauby & Hardy, 2012), pointing out its great utility by comparing it to Hill numbers and IBR. Importantly, however, we do not want to imply that ENS rarefaction is always preferable to the other two families of diversity measures. As a matter of fact, all three families are perfectly suitable representations of a given SAD that carry the same information and allow for conversion between them (Chao et al., 2014; Dauby & Hardy, 2012).

Although we decompose the observed diversity into distinct components, it is important to realize that the components do not strictly exist or change in isolation from another. For example, more‐individual effects can only occur in the presence of a larger scale SAD, and conversely, no species pool can be maintained without the individuals that populate it. Furthermore, the components do not *cause* the observed species richness but rather they concomitantly go along with it. Despite this mutual dependence, we think that a quantitative dissection is useful from an analytical point of view, and our approach represents a consistent quantitative framework for the description of multidimensional and scale‐dependent diversity patterns. Moreover, although our approach is agnostic about mechanism per se, it can provide the empirical patterns to test causal hypotheses of biodiversity variation.

Our approach is applicable for data sets that contain community composition with species abundances that were obtained using standardized sampling procedures. Specifically, we require individual counts and therefore the method is not applicable to indirect proxies of abundance such as biomass or percent cover. If sampling effort varies from sample to sample, the *N*‐effect does not only reflect natural variation in community abundance but also the variable sampling effort. Furthermore, like most approaches to measuring diversity, we assume that the samples are random subsets of the species pool (i.e. independence of all individuals in the sample), and that all species have the same detection probability. Whenever these assumptions are violated, sample‐based rarefaction approaches may be more appropriate (e.g Gotelli & Colwell, 2001; McGlinn et al., 2019).

Here, we modeled the components of diversity as a linear function with latitude. However, the method can be used to explore more complex, nonlinear functional forms. For example, it may be possible that a linear gradient at the species richness level is actually the compound result of nonlinear underlying components, or vice versa. Furthermore, when data are available at multiple spatial grains, this method can be extended to quantify and dissect the effect of spatial aggregation. To do this, we would analyze how the SAD‐component changes between a larger and a smaller scale. Since any scale dependence of SADs are caused by nonrandom spatial distributions, SAD effects between scales can be interpreted as an effect of spatial aggregation (Engel et al., 2021; Olszewski, 2004).

In conclusion, we have shown how the ENS transformation of the rarefaction curve can contribute to quantifying the components underlying diversity gradients. Looking ahead, we think that the ENS curve will be a useful tool for the resolution of a number of open questions regarding the complex interactions between aspects of diversity and sampling. Not only can it shed light onto aspects of evenness in the presence of sampling effects, but when applied across spatial scales, it promises comparable insights into the spatial structure of regionally common and rare species. We hope these approaches will pave the way for a deeper understanding of the patterns and potential drivers of biodiversity change along natural and anthropogenic gradients.

## AUTHOR CONTRIBUTIONS

**Thore Engel:** Conceptualization (lead); formal analysis (lead); methodology (lead); visualization (lead); writing – original draft (lead). **Shane A. Blowes:** Conceptualization (supporting); formal analysis (supporting); methodology (supporting); supervision (supporting); writing – original draft (supporting); writing – review and editing (equal). **Daniel J. McGlinn:** Conceptualization (supporting); methodology (supporting); writing – original draft (supporting); writing – review and editing (equal). **Nicholas J. Gotelli:** Conceptualization (supporting); methodology (supporting); writing – original draft (supporting); writing – review and editing (equal). **Brian J. McGill:** Conceptualization (supporting); methodology (supporting); writing – original draft (supporting); writing – review and editing (equal). **Jonathan M. Chase:** Conceptualization (supporting); formal analysis (supporting); methodology (supporting); supervision (lead); writing – original draft (supporting); writing – review and editing (equal).

## CONFLICT OF INTEREST

The authors declare that there are no conflicts of interest.

## Supporting information


Fig S1

Fig S2

Fig S3

## Data Availability

The data used in this study are already entirely in the public domain. The Gentry forest plot data set (Gentry, 1988, Phillips & Miller, 2002) is accessible through the Botanical Information and Ecology Network (BIEN, https://bien.nceas.ucsb.edu/bien/). The RLS data set of global reef fish abundance and biomass (Edgar et al., 2020; Edgar & Stuart‐Smith, 2014) is accessible through the Australian Ocean Data Network (AODN, https://portal.aodn.org.au/). We also archived the data in Dryad under https://doi.org/10.5061/dryad.rjdfn2zdt.
