# Supplementary figures and images for: How does variation in total and relative abundance contribute to gradients of species diversity?
Source: Ecol Evol. 2022 Aug 17;12(8):e9196. doi: 10.1002/ece3.9196 (PMC9382643; doi:10.1002/ece3.9196)

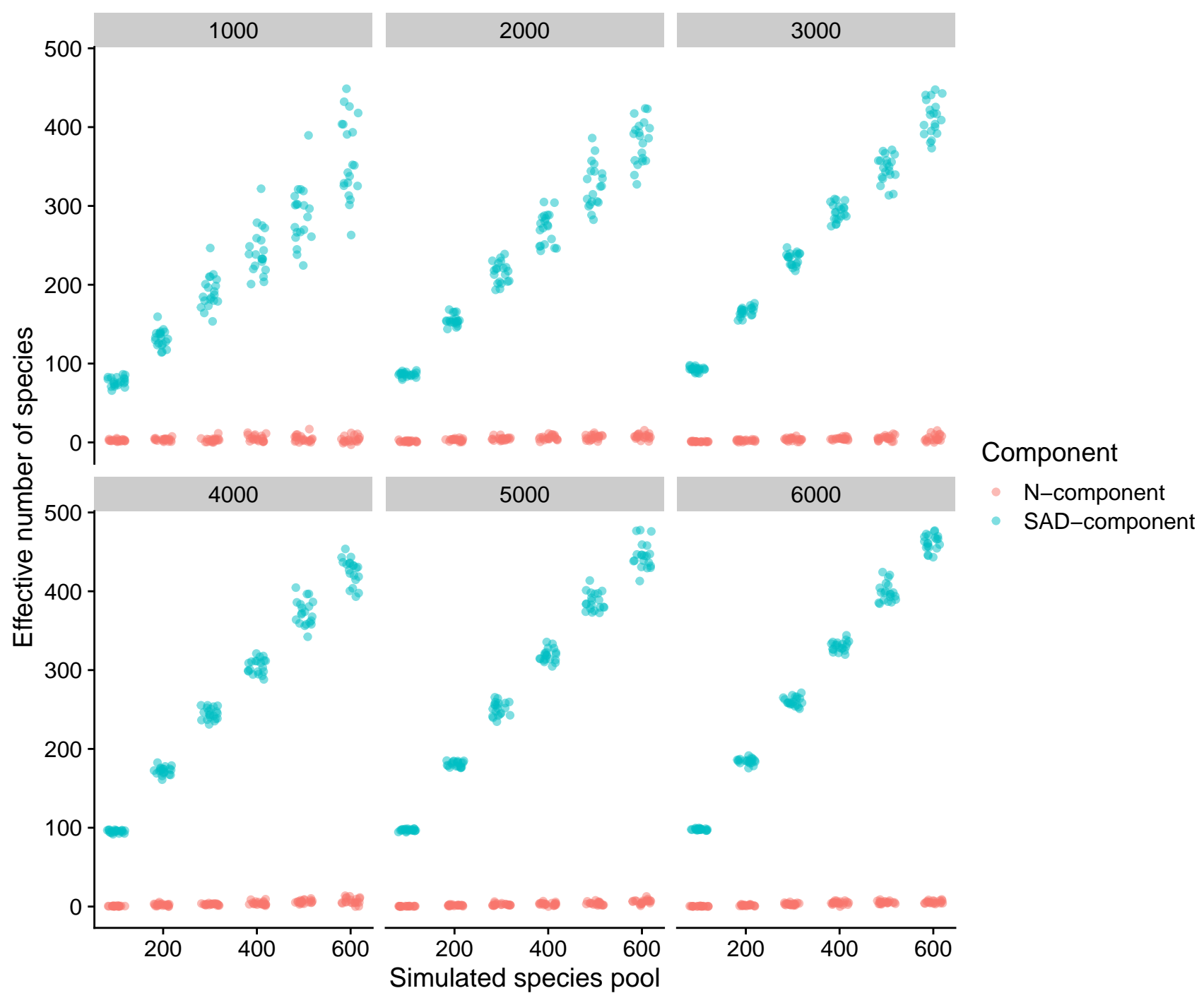

Supplement: Supplementary file 1 — Fig S1 [file ECE3-12-e9196-s003.pdf]

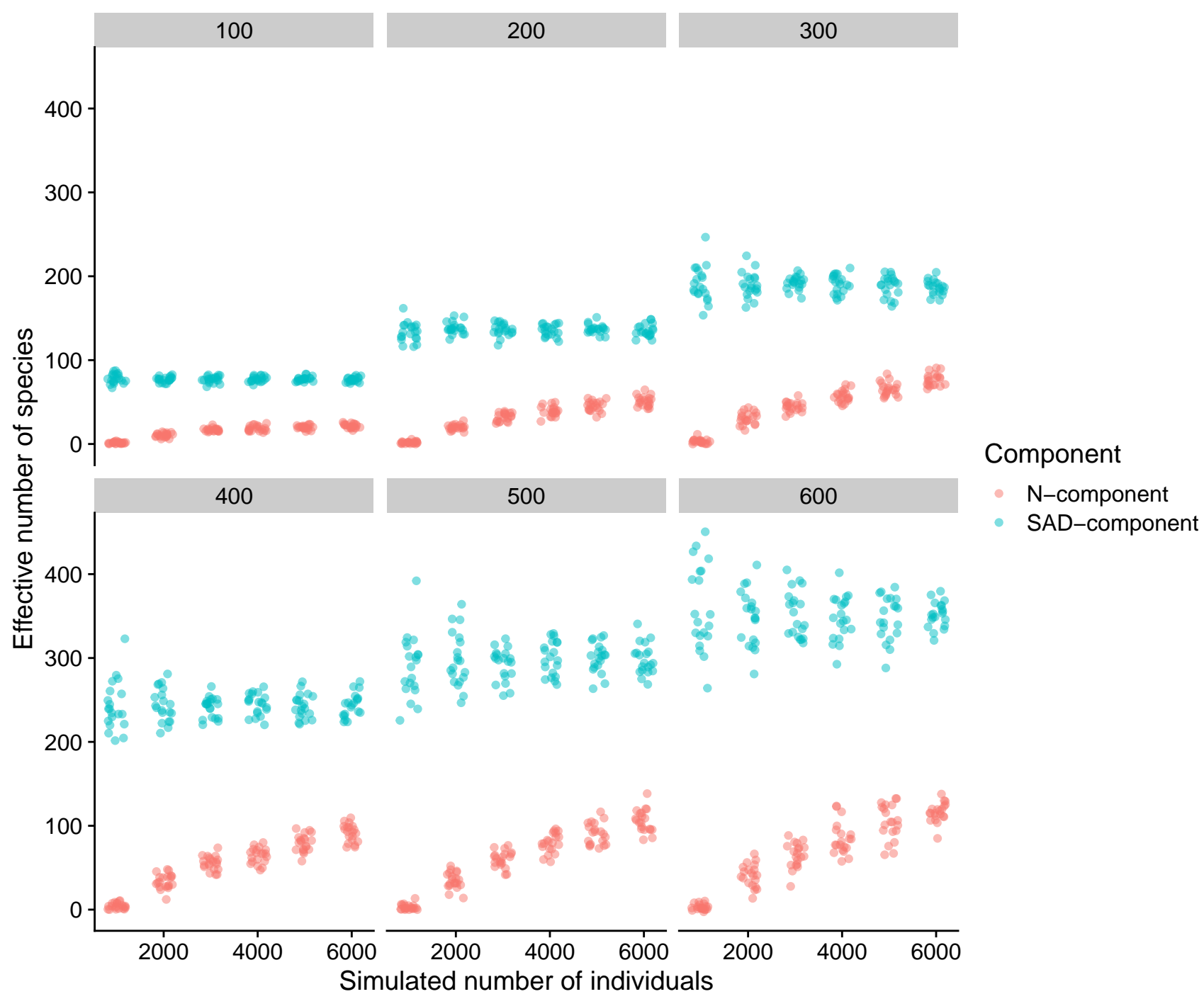

Supplement: Supplementary file 2 — Fig S2 [file ECE3-12-e9196-s001.pdf]

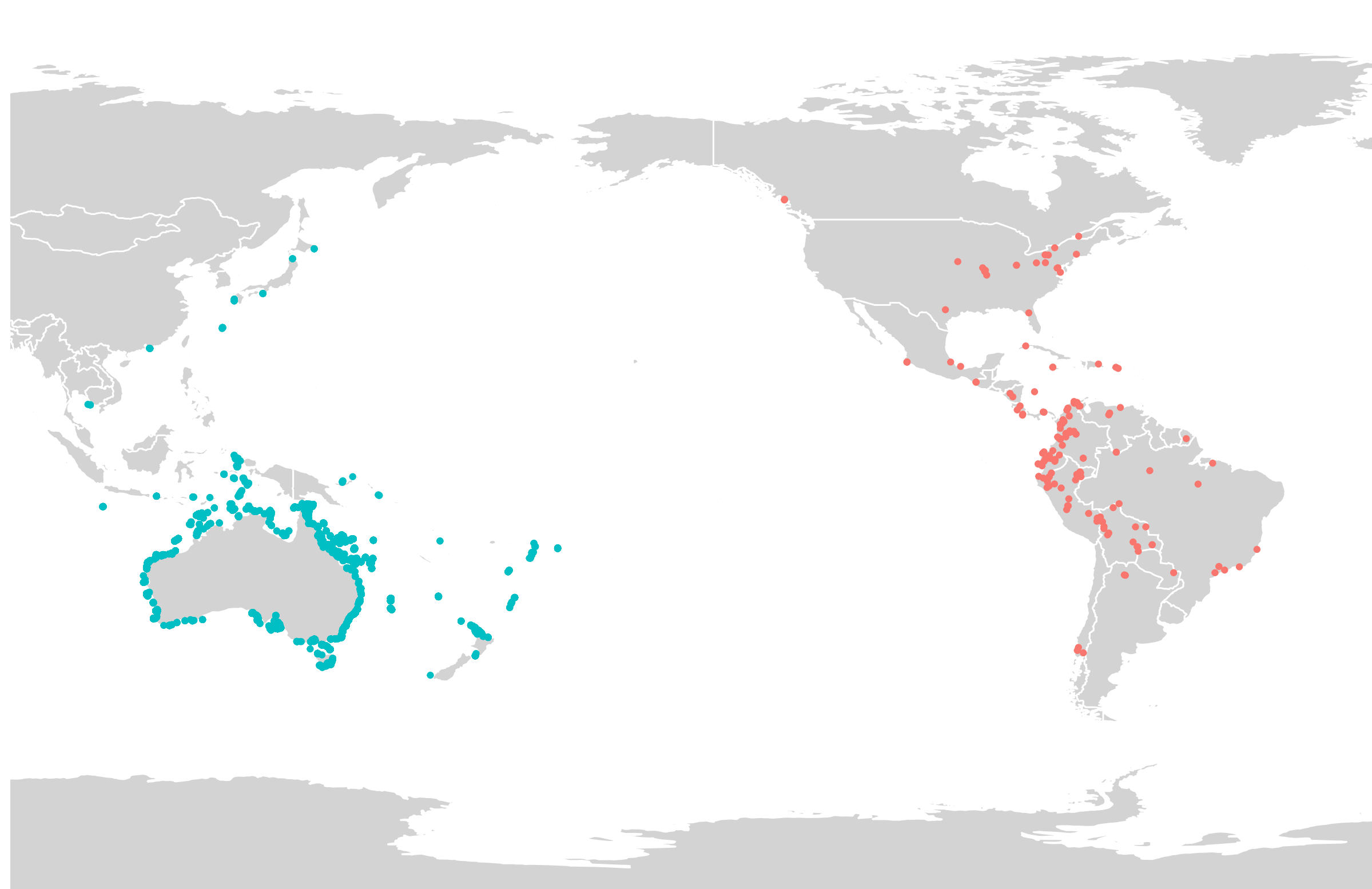

dataset • gentry • RLS

Supplement: Supplementary file 3 — Fig S3 [file ECE3-12-e9196-s002.pdf]
